# Editorial: Inclusive positive psychology interventions for vulnerable communities: bridging gaps and fostering resilience

**DOI:** 10.3389/fpsyg.2026.1998311

**Published:** 2026-10-15

**Authors:** He Bu, Wenjie Duan, Yanlin Zhou, Yihan Wu

**Affiliations:** 1 School of Social and Public Administration, East China University of Science and Technology, Shanghai, China; 2 Department of Psychology, University of Warwick, Coventry, United Kingdom; 3 Max Planck Institute for Psycholinguistics, Nijmegen, Netherlands; 4 Sociology and Social Security, Nanjing Normal University, Nanjing, China

**Keywords:** cultural adaptation, intervention, positive psychology, resilience, vulnerable communities

## Vulnerability is contextual, not dispositional

Positive psychology has expanded the focus of psychology from pathology to strengths and flourishing. But a language of strengths can be exclusionary if adversity becomes a deficit of mindset. Vulnerability is not a static quality of particular people but a situational condition that arises from illness, caregiving responsibilities, cultural marginalization, lack of resources, and institutional exclusion. This Research Topic consists of 10 contributions that cover health, family, educational, cultural, rural, and environmental contexts. Together, they offer more than a catalog of correlates and interventions; they argue that inclusive positive psychology should move beyond simply helping people adapt positively to adversity and start to redesign the relational, cultural, technological, and institutional conditions under which flourishing becomes possible (Kern et al., 2020; Lomas et al., 2021; Prilleltensky, 2012).

The Research Topic also expands the definition of intervention knowledge. Several contributions do not test a manualized positive psychology intervention (PPI) but instead uncover leverage points in living systems. This difference is important because concentrating only on programs delivered to individuals can obscure whether the context provides the opportunity for new skills to be enacted. A strength cannot function as a resource if institutions prevent its use, if cultural meanings negate it, or if caregiving demands consume the time needed to maintain it. An ecological reading places mechanisms, implementation contexts, and structural constraints within the same explanatory frame.

## Positive resources do not operate uniformly

Several studies challenge the assumption that more positivity is invariably better. In a network of 1,271 junior high school teachers, positive emotion was central, hope bridged goal-oriented pathways and professional commitment, and self-efficacy and resilience occupied distinct positions (Zhang et al.). Among 830 university students, positive solitude was associated with life orientation through self-esteem, with hope strengthening this pathway (Peng and Li). Perceived parental encouragement was positively associated with self-efficacy and resilience among 185 Chinese students, but family conflict weakened its association with resilience (Wu et al.). A mixed-methods study of people with diabetes distinguished adaptive acceptance from passive surrender and identified social support, self-efficacy, positive mindset, and altruism as interacting resources (Cheng et al.). Finally, in a small exploratory study, efficacy and resilience were associated with environmental action, whereas optimism could reduce perceived urgency (Eid et al.). These findings favor mechanism-specific, context-sensitive configurations over a universal package of positive traits.

## Resilience emerges through multisystemic processes

The Research Topic also shifts attention from resilience as an individual trait to the multisystemic processes through which people and their environments interact (Ungar and Theron, 2020; Ungar et al., 2023). Among 458 members of four ethnic groups with endangered scripts, perceived cultural continuity was associated with more coherent self-schemas, higher self-esteem, and lower distress (Zhang and Li). In five rural tourism communities, participation was linked to residents' sense of gain, happiness, and security through collective psychological ownership, and the association between participation and ownership was stronger where government trust was higher (Yang et al.). Interviews with 16 full-time caregivers of autistic children likewise traced successful adaptation to self-regulation, diverse support networks, and proactive community engagement across individual, family, and societal levels (Chen L. et al.). Culture and community, therefore, are not layers added after an intervention is designed; they are components of the intervention itself. Moreover, resilience emerges through reciprocal interactions among personal agency, relationships, cultural meanings, community resources, services, and institutions, rather than residing solely with individuals.

## Intervention evidence is promising but incomplete

Direct intervention evidence remains limited but informative. A randomized study enrolling 130 childbearing-age patients with breast cancer found that a PERMA-based program improved psychosocial adjustment, self-concept, interpersonal relationships, social support, and posttraumatic growth, with between-group differences maintained at 6 months (Chen H. et al.). A systematic review identified nine experimental family-resilience interventions for families of autistic children, with eight reporting medium-to-large improvements, although studies were predominantly Western, in-person, and mother-focused (Guo et al.). These findings align with wider evidence that non-Western PPIs may be beneficial, while study quality and cultural fit complicate interpretation (Hendriks et al., 2018). The remaining contributions identified mechanisms, boundary conditions, and intervention targets, and they should not be read as tests of PPI efficacy.

## Toward culturally adaptive intervention ecosystems

The next frontier is not merely culturally adapted intervention, but culturally adaptive intervention: modular systems that can respond to changing identities, family structures, illness stages, and community conditions. Sequential multiple-assignment trials, micro-randomized trials, N-of-1 designs, experience sampling, and dynamic network models could investigate which component works, for whom, when, and under what conditions. Yet personalization must not become another form of individualization. Communities should help define flourishing, select outcomes, govern data, and interpret findings. Specifically, the unit of intervention should expand from individuals to caregiving networks, schools, clinical systems, community organizations, and local policy.

This shift will require new research infrastructure. Rather than treating cultural group membership as a static moderator, studies could model changing exposure to adversity, access to resources, and social position over time. Participatory systems mapping and agent-based simulation could identify leverage points before trials, while realist and implementation evaluations could explain how interventions travel across settings. Moreover, policy and natural experiments could test whether changes in service access, language protection, caregiver support, or participatory governance alter psychological outcomes. This agenda would make context measurable without reducing culture to a demographic covariate.

Evaluation must also move beyond average effects. Trials should preregister heterogeneity, reversals, and potential harms, including self-blame, stigma, loss of adaptive urgency, and unequal digital access. Outcomes should extend beyond private wellbeing to cultural continuity, collective efficacy, participation, resource accessibility, institutional fairness, trust, and environmental action. Responsible digital and AI-supported PPIs may offer multilingual, low-bandwidth, hybrid support and real-time tailoring, but only with privacy protection, bias auditing, human oversight, and community governance. Their success should be judged not simply by engagement, but by equitable reach, community trust, durable collective agency, and stronger supportive systems.

This Research Topic thus changes the central question. The task is not to make so-called vulnerable people more positive, but to co-create conditions in which diverse communities can flourish without denying adversity, transferring structural responsibility to individuals, or sacrificing cultural difference.
